# The late 1970s: a lull in the action on kuru

**DOI:** 10.1098/rstb.2008.4033

**Published:** 2008-11-27

**Authors:** Phillip I. Tarr

**Affiliations:** Division of Gastroenterology, Edward Mallinckrodt Department of Pediatrics, Washington University School of MedicineCampus Box 8208, 660 South Euclid Avenue, St Louis, MO 63110, USA

The late 1970s were a relatively quiescent period in kuru history. The disease's clinical course ([Bibr bib11][Bibr bib27][Bibr bib1][Bibr bib6][Bibr bib13]), geographical epidemiology (Alpers 1965,1979) and dissemination by endocannibalism (Mathews *et al.* 1968) had been well described in the prior two decades. Seminal animal experiments had demonstrated the transmissibility of long-incubating neurological illness ([Bibr bib12][Bibr bib10]). Whole cohorts of Fore children born after certain infection-spreading mortuary practices were abandoned in the late 1950s would, for the first time in possibly a century, survive to adulthood without dying of kuru ([Bibr bib14][Bibr bib15]). Most tellingly, in each successive year, the median ages of new patients increased by approximately 1 year, a correlation consistent with interdicted transmission. The elusive infective entity appeared less likely to contain DNA or RNA even as molecular genetic techniques for finding nucleic acid improved ([Bibr bib10][Bibr bib16]), but because the Fore no longer faced extermination by kuru, and because community-acquired spongiform encephalopathies were not emerging in other populations except for rare iatrogenic transmissions of Creutzfeldt–Jakob disease, investigative nihilism set into the field of ‘slow agent’ research.

I had an opportunity as a medical student to perform kuru fieldwork for five months in 1979 under the direction of Michael Alpers. I was charged with finding every person living with confirmed or putative kuru in the Eastern Highlands, using Alpers' guidelines (Alpers 1964). Field assistants Anua Senavaiyo, Auyana Winagaiya and Igana Alesagu from the Papua New Guinea (PNG) Institute of Medical Research (IMR) in Goroka took me to each of the 23 kuru patients known to them in that interval. Our goal was to confirm or refute the epidemiological model that the kuru agent had been transmitted to children of both genders, and to girls and women older than approximately 7 years of age, probably owing to age–gender segregation at mortuary gatherings (figure 1). Each of these 23 patients was born before 1954, again confirming the transmission model. I also refined several family histories of intergenerational kuru, analysed childhood infection cohorts and identified an apparently longer incubation period in boys than in girls (Tarr 1980).

However, my chief effort was to interview the relatives and acquaintances of kuru decedents in the ‘Book’. The Book was the voluminous hard copy line listing of all kuru cases by village from 1957 onwards. Its major contributors in the early years were Patrol Officer Jack Baker (a particularly important contributor), Carleton Gajdusek, Vin Zigas and Alpers, who, in 1970, computerized these data at the NIH. Other contributors included Alex Nilsson, John Mathews, Richard Hornabrook and others from the IMR, kiaps (patrol officers), and many local and visiting doctors, students and missionaries. Steve Ono and Judy Farquhar entered the data from the field into the NIH mainframe computer and generated updated paper copies periodically (figure 2). This systematic collection of the estimated year of birth, symptom onset, death or ‘recovery’ of every definite, putative or refuted case of kuru (Alpers & Gajdusek 1965) represents ‘shoe leather’ epidemiology in every sense of the term. These data are as unusual as they are important. I know of no other epidemic where every case has been meticulously documented over a half century, or any disease disappearance so thoroughly chronicled. The MRC, to its credit, has supported continued kuru surveillance, analysis and data archiving.

In the late 1970s, the IMR had limited resources to address the many medical disorders in the newly independent PNG. Michael Alpers, who succeeded Richard Hornabrook as IMR Director in 1977, faced these challenges by seeking new funding, expanding malaria, filariasis and nutrition research, founding IMR branches in the Madang and Southern Highlands Provinces, and supporting extensions of Ian Riley's groundbreaking work on lower respiratory tract infection in Huli children and adults in Tari (Riley *et al.*[Bibr bib21][Bibr bib22][Bibr bib20]). However, Alpers was one of Gajdusek's original collaborators in Okapa and Bethesda, and his family had maintained part-time residence in Waisa throughout the 1960s and 1970s. Alpers therefore knew the value of kuru surveillance, and how to do it. Most importantly, he recognized that without shouldering personal responsibility for the arduous and underfunded kuru fieldwork, a scientifically precious and unique data collection would meet an untimely end. Michael's telephone call to me in Seattle in 1985 from Goroka (where it was 1 o' clock in the morning at the time) requesting clarification on some field notes I had taken 6 years earlier typified his stewardship of these efforts.

Post-1970s kuru science, well described in this issue of the *Transactions*, is highlighted by the perseverance of Stan Prusiner in his refinement of small animal models of scrapie ([Bibr bib7][Bibr bib25]), meticulous chemical methodology ([Bibr bib19][Bibr bib9]) and identification of PrP (Basler *et al.* 1986), and the thorough correlations of John Collinge and his group ([Bibr bib18][Bibr bib24]) between host genotype and prion disease expression, thereby explaining the epidemiology of kuru and other human transmissible encephalopathies. However, the people of the Eastern Highlands remain the underappreciated heroes of this saga. Even though not a single one would plausibly benefit from this research, patients and their relatives unfailingly cooperated with kuru investigators for over a half century. Had the people of the kuru-affected region been less tolerant of the repeated and no doubt baffling studies by outsiders, the concept of transmissible, long-incubating, human encephalopathies would have been accepted tardily, if at all, by clinicians, scientists, governments and industry. Such a delay would have further stalled recognition that food of bovine origin could transmit variant Creutzfeldt–Jakob disease (vCJD), and magnified and prolonged the 1990s vCJD epidemic, since so many people in the United Kingdom are at genetic risk because their human prion protein has a methionine at position 129 (Windl *et al.* 1996). Alpers' continuity, personality and standards facilitated their cooperation, but in the final analysis it was the enduring altruism of the Fore people and their neighbours in the Eastern Highlands of PNG that enabled knowledge to emerge from the kuru tragedy. Kuru sufferers deserve the gratitude of people worldwide.

Kuru is, or will soon be, extinct (Alpers & Kuru Surveillance Team 2005). The South Fore and their neighbours are not. This story has taught us much about scientific inquiry, and about citizenship.

## Figures and Tables

**Figure 1:**
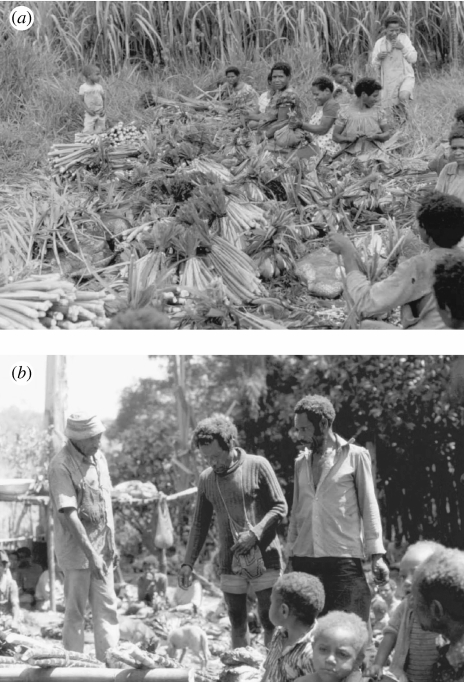
**) Women and children of both genders at funeral feast (29 July 1979) of a woman who died of kuru at Anumpa 2 days before. (**) Boys and men congregating at same feast.

**Figure 2:**
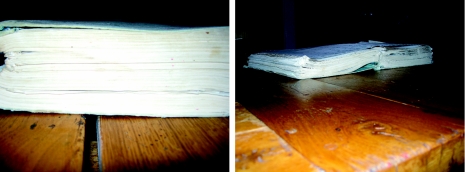
The Kuru Book (1979 version): hard copy line listing of all patients by village.
